# Linear epitopes of PRRSV-1 envelope proteins ectodomains are not correlated with broad neutralization

**DOI:** 10.1186/s40813-024-00393-7

**Published:** 2024-10-21

**Authors:** Jaime Castillo-Pérez, Francisco Javier Martínez-Lobo, Raquel Frómeta, José María Castro, Isabel Simarro, Cinta Prieto

**Affiliations:** 1 Animal Health Department, Faculty of Veterinary Medicine, Universidad Complutense de Madrid, Madrid, Spain; 2 Animal Science Department, School of Agrifood and Forestry Engineering and Veterinary Medicine, University of Lleida, Lleida, Spain

**Keywords:** Porcine reproductive and respiratory syndrome virus, Linear neutralizing epitopes, Broadly reactive neutralizing antibodies

## Abstract

**Background:**

Neutralizing antibodies against PRRSV are capable of conferring protection against viral reinfection, but they tend to be strain specific and usually have poor cross-reactivity. Nonetheless, it has been described that there are individuals capable of efficiently neutralizing viruses of different origin, so it is expected that there are conserved neutralizing epitopes relevant for broad neutralization. However, although immunodominant regions and neutralizing epitopes have been described in different envelope proteins, their role in broad neutralization is unknown. The main objective of this study was to determine whether the linear epitopes existing in the ectodomains of PRRSV envelope proteins play a role in cross-neutralization.

**Results:**

A pepscan analysis was carried out using synthetic peptides against the ectodomains of PRRSV envelope proteins and PRRSV-hyperimmune sera of different cross-reactivity. The results obtained confirm the existence of antigenic regions in the ectodomains of the GP2, GP3, GP4 and GP5 that tend to be relatively conserved among different PRRSV isolates. Nonetheless, these antigenic regions have poor immunogenicity since they are only recognized by a limited number of sera. Furthermore, no differences were found between the reactivity of sera with broad cross-neutralization capacity and sera with poor heterologous neutralization activity, which indicate that linear epitopes existing in the ectodomains of PRRSV envelope proteins are not relevant for the development of broadly reactive neutralizing antibodies. Subsequently, some selected peptides were used in competition assays with the virus for binding to the cell receptors and in seroneutralization inhibition assays by incubation with hyperimmune sera. Firstly, some peptides that interfere with virus infectivity were identified in competition assays, but only in the case of one viral isolate, which points to the possible existence of a strain-dependent inhibition. However, the results of the seroneutralization inhibition assay indicate that, under the conditions of our study, none of the peptides used was capable of inhibiting virus neutralization by the hyperimmune sera.

**Conclusions:**

The results obtained indicate that the linear peptides analyzed in this study do not play a major role in the induction of broadly reactive neutralizing antibodies, which could probably depend on conformational neutralizing.

**Supplementary Information:**

The online version contains supplementary material available at 10.1186/s40813-024-00393-7.

## Background

Porcine reproductive and respiratory syndrome (PRRS) is one of the most important infectious diseases that affect the pig industry worldwide. It is characterized by severe reproductive disorders in sows and respiratory distress in young growing and finishing pigs, predisposing them to secondary infections associated with the porcine respiratory disease complex [1]. The etiological agent of PRRS is PRRS virus (PRRSV), an enveloped virus that belongs to the genus *Betaarterivirus* within the *Arteriviridae* family in the *Nidovirales* order [2].

Arteriviruses share common properties, including the ability to replicate in monocyte-macrophage cells and the capacity to cause persistent infections, although genomic variability is considered the hallmark of this family of viruses [3]. Thus, PRRSV genomic heterogeinity is so remarkable that PRRSV isolates are currently classified into two different species: *Betaarterivirus suid 1*, known as PRRSV-1 (former genotype 1), which are distributed mainly in European countries, and *Betaarterivirus suid 2*, known as PRRSV-2 (former genotype 2), widely spread in [4] American and Asian countries [2]. Furthermore, genomic variability is also observed within each specie and several lineages and subtypes have been described [4–6].

The PRRSV genome consists of a single-stranded positive-sense RNA molecule that varies from 15.1 to 15.5 kb in length. A total of ten overlapping open reading frames (ORFs) that undergo subgenomic mRNA generation through a mechanism of discontinuous transcription have been described to date [7].

At the 5’ terminus of the genome, overlapping ORFs 1a and 1b comprise about 80% of the viral genome. These ORFs encode two polyproteins that, upon cleavage by viral proteases, yield 12 to 14 non-structural proteins involved in viral replication [3, 8]. In contrast, the 3’ terminus encompasses eight overlapping ORFs encoding eight essential structural proteins, with four being glycosylated and four non-glycosylated. Notably, the N protein, derived from ORF7, is particularly abundant in infected cells, comprising 20–40% of the virion’s protein content [9, 10].

The glycosylated proteins of PRRSV GP2, GP3 and GP4 interact themselves and with protein E, forming heterotetramers within infected cells. These complexes are essential for appropriate protein processing [11]. While these heterotetramers are sparsely represented in the virion and not directly involved in viral progeny release, their importance for virus infectivity is evident through their interaction with the CD163 receptor, recognized as a critical determinant of infection susceptibility [11, 12].

GP5, the predominant envelope protein, forms disulfide-linked heterodimers with the M protein via disulfide bridges, exerting an important role in viral assembly and progeny release. The GP5-M complex is further implicated in viral entry, engaging with potential virus receptors such as heparin sulfate molecules (M protein) and sialoadhesin or CD169 (GP5). These interactions suggest their involvement in the attachment of the virus to target cells [12].

Additionally, other forces lead to the interaction between all PRRSV structural proteins. For instance, GP4’s interaction with GP5 initiates an intricate cascade of interactions between GP5-M heterodimers and GP2-GP3-GP4-E heterotetramers, culminating in a complex that includes all structural proteins except for N [11]. While interactions with other receptors facilitate infection, it is been stablished the interaction with CD163 is necessary for the infection, as confirmed by studies involving genetically modified pigs lacking specific CD163 domains [13].

Due to the influence that neutralizing antibodies (NAs) can have in protecting animals against reinfections, the search for antigenic regions (ARs) that may potentially contain neutralizing epitopes (NEs) has become a priority. The result of these investigations has been the discovery of potentially NEs in almost all structural proteins of the virus. Thus, NEs have been described in GP2 [14], GP3 [14, 15], GP4 [15–17], GP5 [15, 18–20], and the M protein [15, 21, 22]. The first NE identified is NE located in a hypervariable region of the ectodomain of PRRSV-1 GP4 [16]. Shortly after, a second NE was described in the GP5 ectodomain of PRRSV-2 [18, 20] and PRRSV-1 [19]. However, the presence of this NE in PRRSV-1 has been questioned in subsequent studies [14]. More recently, the existence of three additional NEs have been determined in PRRSV-1, one in GP3, between positions 61 and 72, and two in GP2, between positions 37 and 48 and positions 117 and 128 [14]. Finally, Trible et al. [22] reported that a deletion in position 10 of a PRRSV-2 M protein ectodomain confers resistance to neutralization, indicating that this position could play a key role for PRRSV neutralization.

The presence of all these NEs in PRRSV virion contrasts with both the poor development of NAs after infection and the low cross-reactivity between isolates reported in cross-neutralization studies [17, 23], which question both their immunogenicity and the conservation of the sequence of these NEs between isolates. Nonetheless, some studies indicate that there is a small proportion of sera, both from experimental infections and from the field, capable of effectively recognizing heterologous strains in in vitro cross-neutralization assays [23, 24]. This finding points to the existence of conserved NEs among isolates, which can be recognized by at least some sera. The same phenomenon has been described for other viruses, such as Human Immunodeficiency Virus (HIV) or Influenza Virus [25, 26] and has led to the theory that these poorly immunogenic NEs could play a relevant role in protection against viral infections caused by highly variable viruses.

Therefore, the aim of this study was to determine whether the linear epitopes previously described in the literature in the ectodomains of the PRRSV-1 envelope proteins exist across different PRRSV-1 isolates and whether they are differentially recognized by PRRSV-1 hyperimmune sera with different broad neutralization capabilities with the ultimate goal of identifying linear NE which could be relevant for cross-neutralization.

## Methods

### PRRSV isolates and hyperimmune monospecific sera

Three PRRSV-1 isolates were used in this study (Table 1). The sequences of ORF2 to ORF6 of each isolate were obtained and used to predict the corresponding amino acid sequences, as previously described [23, 27].

A total of 32 porcine PRRSV monospecific hyperimmune sera from a previously existing collection were used in this study (Table 2). Eleven of these sera were monospecific hyperimmune sera that had previously demonstrated a high capacity to neutralize in vitro heterologous isolates in cross-neutralization assays, exhibiting high breadth and potency [26]. Another eleven monospecific hyperimmune sera exhibited a limited ability to neutralize heterologous isolates in cross-neutralization assays. Finally, ten PRRSV-negative sera were used to determine the nonspecific reactivity of the technique.

**Table 1:** PRRSV isolates used in this study

Isolate	Specie	Country of isolation	Year of isolation	Genbank accession no. ORFs2-6
EU-14	PRRSV-1	Hungary	2007	OP643815;JF730917;JF730956;JF730995;PP035730
EU-21	PRRSV-1	United Kingdom	2005	OP643818;PP035698;PP035706;PP035714;PP035733
EU-24	PRRSV-1	Italy	2007	OP643820;PP035700;PP035708;PP035176;PP035738

**Table 2:** Characteristics of the hyperinmmune monospecific sera used in this study

Serum	Isolate used for immunization	Cross neutralization ability	NA titer (log_2_)			
			Sp-3	EU-21	EU-24	EU-14
Sp-2-1	Sp-2	H^a^	2.0	3.0	3.0	3.0
Sp-2-2	Sp-2	H	4.0	2.0	2.0	5.0
Sp-3-1	Sp-3	H	7.0	4.0	3.0	4.0
Sp-3-2	Sp-3	H	6.0	5.0	5.0	3.0
Sp-5-1	Sp-5	H	5.0	2.0	4.0	1.0
Sp-28-1	Sp-28	H	3.0	3.0	1.0	3.0
EU-9-1	EU-9	H	3.0	3.0	3.0	3.0
EU-14-1	EU-14	H	5.0	4.0	4.0	7.0
EU-14-2	EU-14	H	4.0	3.0	3.0	7.0
EU-21-1	EU-21	H	3.0	5.0	5.0	5.0
EU-21-2	EU-21	H	4.0	6.0	3.0	5.0
Sp-2-3	Sp-2	L^b^	3.0	1.0	1.0	3.0
Sp-3-3	Sp-3	L	5.0	1.0	0.1^d^	1.0
Sp-5-2	Sp-5	L	2.0	0.1	0.1	0.1
EU-9-2	EU-9	L	2.0	0.1	0.1	0.1
EU-11-1	EU-11	L	0.1	0.1	0.1	1.0
EU-11-2	EU-11	L	1.0	0.1	0.1	0.1
EU-14-3	EU-14	L	1.0	2.0	1.0	6.0
EU-14-4	EU-14	L	1.0	0.1	1.0	7.0
EU-23-1	EU-23	L	1.0	1.0	0.1	2.0
EU-24-1	EU-24	L	0.1	0.1	6.0	1.0
EU-24-2	EU-24	L	0.1	0.1	6.0	0.1
Sp-2-2-0	PRRSV negative	N^c^	0.1	0.1	0.1	0.1
Sp-3-1-0	PRRSV negative	N	0.1	0.1	0.1	0.1
Sp-3-2-0	PRRSV negative	N	0.1	0.1	0.1	0.1
Sp-5-1-0	PRRSV negative	N	0.1	0.1	0.1	0.1
Sp-5-2-0	PRRSV negative	N	0.1	0.1	0.1	0.1
EU-9-2-0	PRRSV negative	N	0.1	0.1	0.1	0.1
EU-11-1-0	PRRSV negative	N	0.1	0.1	0.1	0.1
EU-21-1-0	PRRSV negative	N	0.1	0.1	0.1	0.1
EU-21-2-0	PRRSV negative	N	0.1	0.1	0.1	0.1
EU-24-2-0	PRRSV negative	N	0.1	0.1	0.1	0.1

^a^H: sera with good breadth and potency, capable of neutralizing heterologous PRRSV isolates

^b^L: sera with low capacity for heterologous neutralization

^c^N: PRRSV-negative sera

^d^: 0.1 (log2) indicates absence of neutralization

### Pepscan analysis

For the identification of reactive epitopes, a Pepscan analysis was conducted using overlapping peptides in a peptide ELISA set as previously described [28]. The peptides were designed based on the predicted amino acid sequences of GP2, GP3, GP4, GP5, and M proteins. A consensus prediction of membrane protein topology was estimated using TopCons computer program [29] and only the ectodomains of the proteins were considered. Sets of overlapping dodecapeptides with an offset of 4 and an overlap of 8 amino acid were designed by Peptide Library Design and Calculator Tool software (Sigma-Aldrich Co, St. Louis, MO, USA). A total of 422 biotinylated peptides (BioTides) were chemically synthesized (JPT Peptide Technologies GmbH, Berlin, Germany) to allow immobilization to streptavidin-coated 96-well plates for immunological assays. The peptides were resuspended in dimethyl sulfoxide (DMSO) at a concentration of 500 µg/µL and stored at -80 °C until used in the ELISA assays.

Streptavidin-coated 96-well plates (Thermo Scientific™ Nunc™ Immobilizer™ Streptavidin Plates, Thermo Fisher Scientific, Waltham, MA, USA were coated with 0.1 µg/well of the corresponding peptide diluted in PBS with 0.05% Tween-20 and 40% DMSO. All sera were diluted 1:200 in washing solution (PBS with 0.05% Tween-20) and incubated on the peptide-coated plates for 1 h at room temperature. After incubation, the plates were washed with washing solution and incubated with peroxidase-conjugated rabbit-anti-swine polyclonal antibodies (Sigma-Aldrich Co, St. Louis, MO, USA) diluted 1:40.000 in washing solution for 1 additional hour. Then, the plates were washed again, and the reaction developed with a substrate solution of tetramethylbenzidine and H_2_O_2_ (TMB, Thermo Fisher Scientific, Waltham, MA, USA)). After 10 min, the reaction was stopped with 1 M H_2_SO_4_, and the optical density at 450 nm (OD450) was measured.

The analysis of the obtained optical density values was conducted according to the procedure described by Vanhee et al. (2011) [14]. Thus, the value of each serum against a specific peptide were expressed in relation to the mean value obtained with negative sera against the same peptide (OD450 s/n). Additionally, the OD450 s/n value for all peptides of the same protein was calculated (average OD450 s/n). Finally, if the OD450 s/n value of a serum against a peptide was more than two times higher than the mean value of all peptides of the protein (OD450 s/*n* > 2x average OD450 s/n), the signal was considered specific.

### Synthesis of recognized ectodomain peptides

A total of 27 peptides of the 422 used in the study were selected for functional analysis. These peptides were manufactured using solid phase FMOC or BOC chemistry methodologies on a PEG-Polystyrene support resin (Sigma-Aldrich Co, St. Louis, MO, USA) with a purity of 80%. In addition, two irrelevant peptides were included, one from the E2 protein of the classical swine fever virus (CSFV) (KEYSHGLQLNDG) and another consisting of the antibacterial action peptide called pleurocidin, from the *Pseudopleuronectes americanus* (GWGSFFKKAAHL), which acted as negative controls [30].

All peptides were solubilized at a concentration of 5 mg/mL in 0.1 M PBS at pH 7.4, except for those of a hydrophobic nature that were solubilized in DMSO, following the manufacturer’s instructions. All subsequent dilutions of the 5 mg/mL stock were performed using Dulbecco’s Modified Eagle Medium (DMEM) culture medium.

### Cellular receptor competition assay

For the cellular receptor competition assay, 96-well cell culture plates (VWR^®^ Tissue Culture Plate, VWR International, Radnor, PA, USA), were employed. In each well, 10,000 MARC-145 cells were seeded 24 h prior to the assay. Selected peptides were added to four wells at a final concentration of 100 µg/mL. The cells were incubated with the corresponding peptide for one hour at 37 °C. Subsequently, 50 µL of a virus suspension containing a total of 100 TCID_50_ was added, followed by another one-hour incubation at 37 °C.

After this incubation period, the mixture containing the virus and the peptides was carefully removed from the cell monolayer. MARC-145 cells were gently washed with PBS pre-warmed to 37 °C and DMEM culture medium supplemented with 5% Fetal Bovine Serum (FBS) was added. The presence or absence of the cytopathic effect (CPE) characteristic of PRRSV-1 was observed at day six post-infection.

In all plates, DMEM culture medium supplemented with 5% FBS was employed as a negative control and the peptide diluents, i.e. 0.1 M PBS and DMSO, were included to assess potential cellular toxicity or interference with the assay. All assays were performed in duplicate and repeated three times.

### Neutralization-inhibition assay

To determine the effects of peptide addition on the neutralization capacity of the sera, neutralization-inhibition assays were carried out as previously described [31]. Briefly, synthetic peptides (individually or selected mixtures) were added to the serum dilutions at a final concentration of 100 µg/mL and incubated for 1 h at 37 °C. Subsequently, the serum/peptide mixture was incubated for 1 h at 37 °C in a humidified atmosphere with 100 TCID_50_ of one of the PRRSV isolates used in the study. After this time, 100 µL of a cell suspension containing 10,000 cells of the MARC-145 cell line prepared in DMEM supplemented with 10% FBS were added to each well. After 6 days of incubation at 37 °C in an atmosphere with 5% CO_2_, the presence of the characteristic CPE of the PRRSV was determined in each of the wells. All samples were analyzed in duplicate and the NA titer was expressed as the highest dilution of serum that could neutralize the action of the virus in at least one of the two wells used, expressed as logarithm to base two (log_2_). As a control of the neutralizing activity of the sera used and to determine the inhibitory capacity of the peptides used in the study, the seroneutralization (SN) assays were carried out in parallel without the addition of the corresponding peptide. All neutralization-inhibition assays were conducted in duplicate and repeated three times.

### Statistical analysis

Differences in the proportion of sera with broad and poor neutralizing activity that reacted with each peptide were assessed for significance by the two-tailed Fisher’s exact test. In addition, differences in GMT of NAs obtained in neutralization inhibition assay and inhibition capacity of the different peptides were analyzed using Kruskal-Wallis nonparametric and Mann-Whitney U tests.

All statistical tests were carried out with GraphPad Prism software, and results were considered as statistically significant when p value was less than 0.05.

## Results

### Reactivity of the individual sera against the linear peptides of PRRSV-1 envelope proteins ectodomains

None of the peptides used in this study was specifically detected by any of the ten PRRSV negative sera included as controls. On the contrary, up to one hundred and fifty six peptides among those existing in the ectodomains of the structural proteins GP2, GP3, GP4, GP5 and M from the three PRRSV-1 isolates used in this study (i.e. EU-14, EU-21 and EU-24) were recognized by at least one of the monospecific hyperimmune sera tested. The individual reactivity pattern of all sera against the peptides used in this study are available as supplementary data (Supplementary Data). However, only a total of 25 peptides were recognized by four or more hyperimmune sera (Table 3).

#### Peptides recognized in the ectodomain of GP2

Sixteen antigenic peptides were identified in the ectodomain of GP2 in isolates EU-14 and EU-24 and eighteen in the case of isolate EU-21 (Supplementary data). Notably, all peptides detected in the ectodomain of GP2 were recognized by at least one heterologous serum. However, as it can be observed in Table 3, only 2 peptides in EU-21 and EU-24 were recognized by at least 3 different sera. Remarkably, these peptides are the equivalent in EU-21 and EU-24 of two NEs previously described in Lelystad Virus (LV), i.e. GSPSQDGYWSFF and EHSGQAAWKQVV.

In the case of the PRRSV-1 isolate EU-14, the reactivity of the sera against the GP2 peptides used in this study was much higher. Thus, two peptides were recognized by at least 50% of the sera, while the peptide equivalent to that described as a NE in LV (EHSGQAAWKQVV) was recognized by 5 heterologous sera.

Finally, it is remarkable that some peptides in the ectodomain of GP2 were only detected by broadly neutralizing sera. Thus, three of the sera with high breadth and potency reacted against an EU-21 AR not previously described as neutralizing while this AR was not recognized by any of the sera with low cross-reactivity. However, no statistically significant differences were observed between the reactivity of broadly and poor neutralizing heterologous sera (*p* > 0.05).

#### Peptides recognized in the ectodomain of GP3

Eighteen antigenic peptides were identified in the ectomain of GP3 in isolates EU-21 and EU-24 and sixteen in EU-14. However, the number of reactive sera was more variable than in other proteins and it ranged from one single serum to 16 (Supplementary Data, Table 3). A broad recognition was particularly observed in the region corresponding to the NE previously described for this protein in LV (i.e. QAARQRLEPGRN) and in the AR located immediately downstream (Supplementary data). In relation to the NE it is noteworthy that all homologous sera but one (i.e. one of the sera specific for EU-21) recognized at least one peptide in the region of the NE, regardless their cross-reactivity. Even more, the peptide corresponding to the NE in EU-24 was the only peptide recognized by the homologous sera in the ectodomain of this protein. Besides, these peptides were recognized by a significant number of heterologous sera. Although there were no statistically significant differences between the reactivity of sera of different breadth and potency against the GP3 peptides (*p* > 0.05) there was a trend that a greater number of sera classified as highly cross-reactive reacted with the set of peptides related to the NEs described above. Finally, it is remarkable that the region comprised between positions 238–257 in GP3 of EU-24 was recognized by up to eleven sera, both broadly-neutralizing and of poor cross-reactivity. However, the same region was poorly recognized in the other viral isolates.

#### Peptides recognized in the ectodomain of GP4

Twelve, fifteen and thirteen peptides were recognized by at least one serum in GP4 ectodomain of isolates EU-14, EU-21 and EU-24, respectively.

The hypervariable NE previously described in the ectodomain of GP4 [32] was recognized only by the homologous sera in the case of EU-14 and EU-21. Noteworthy, the region equivalent to this epitope was not recognized by any serum in the case of EU-24.

Furthermore, the most frequently recognized peptides in the ectodomain of GP4 were peptides that have not been previously identified as immunogenic. Thus, the peptide ITANVTDESYLY, located downstream of the hypervariable NE in EU-14, was recognized by seven different sera while the peptide AVGTPQYITMTA, also located downstream of the hypervariable NE in EU-21 was recognized by up to nine sera. Finally, in the case of EU-24 the most frequently recognized peptide was peptide TAAAGFLVLQDI, located upstream of the region corresponding to the hypervariable NE, that was recognized by seven sera (Supplementary data).

However, when the recognition pattern of broadly reactive and poorly-cross-reactive sera was studied, no statistically significant differences were found for any of the peptides located in the ectodomain of GP4.

#### Peptides recognized in the ectodomain of GP5

The number of peptides identified as immunogenic in the GP5 ectodomains was lower than in the rest of the glycoproteins analyzed and only seven, six and five peptides were recognized by some of the sera in isolates EU-14, EU-21 and EU-24, respectively (Supplementary data).

The seven immunogenic peptides of the isolate EU-14 were recognized by a variable number of sera (i.e. between 1 and 9). The most reactive peptide corresponds to an epitope previously described as neutralizing [18] and it was recognized by sera of different properties, including homologous sera of broad and poor cross-reactivity and a set of heterologous sera of different specificities and qualities.

In the case of the isolate EU-21 the pattern of recognition was different. A total of six peptides were recognized by a variable number of sera (i.e. between one and eleven). The most immunogenic peptide (i.e. NGDSSTYQYIYN) contained the previously described NE, but it was not recognized by any of the homologous sera. On the contrary, this peptide was recognized by a significant number of heterologous sera of different neutralization capacity (Table 3).

Finally, in the case of the isolate EU-24, five peptides were recognized by between 1 and 9 sera. Similarly to the other two isolates, the most frequently recognized peptide (i.e. KGDSSTYQYIYN) was located in the region corresponding to the previously described NE and it was recognized by a variety of sera, including the two homologous sera included in the study and a significant proportion of heterologous sera of different neutralizing qualities.

Despite the reactivity of the sera with the abovementioned peptides, when the influence of the neutralization properties of the sera on the reactivity against these peptides was analyzed, no significant differences were found between broadly and poorly reactive sera (*p* > 0,05).

#### Peptides recognized in the ectodomain of M protein

Only one peptide was recognized in the ectodomain of the M protein of isolates EU-14 and EU-24 and it was only recognized by a low number of heterologous sera (1 and 2, respectively) (Supplementary data).

**Table 3:**
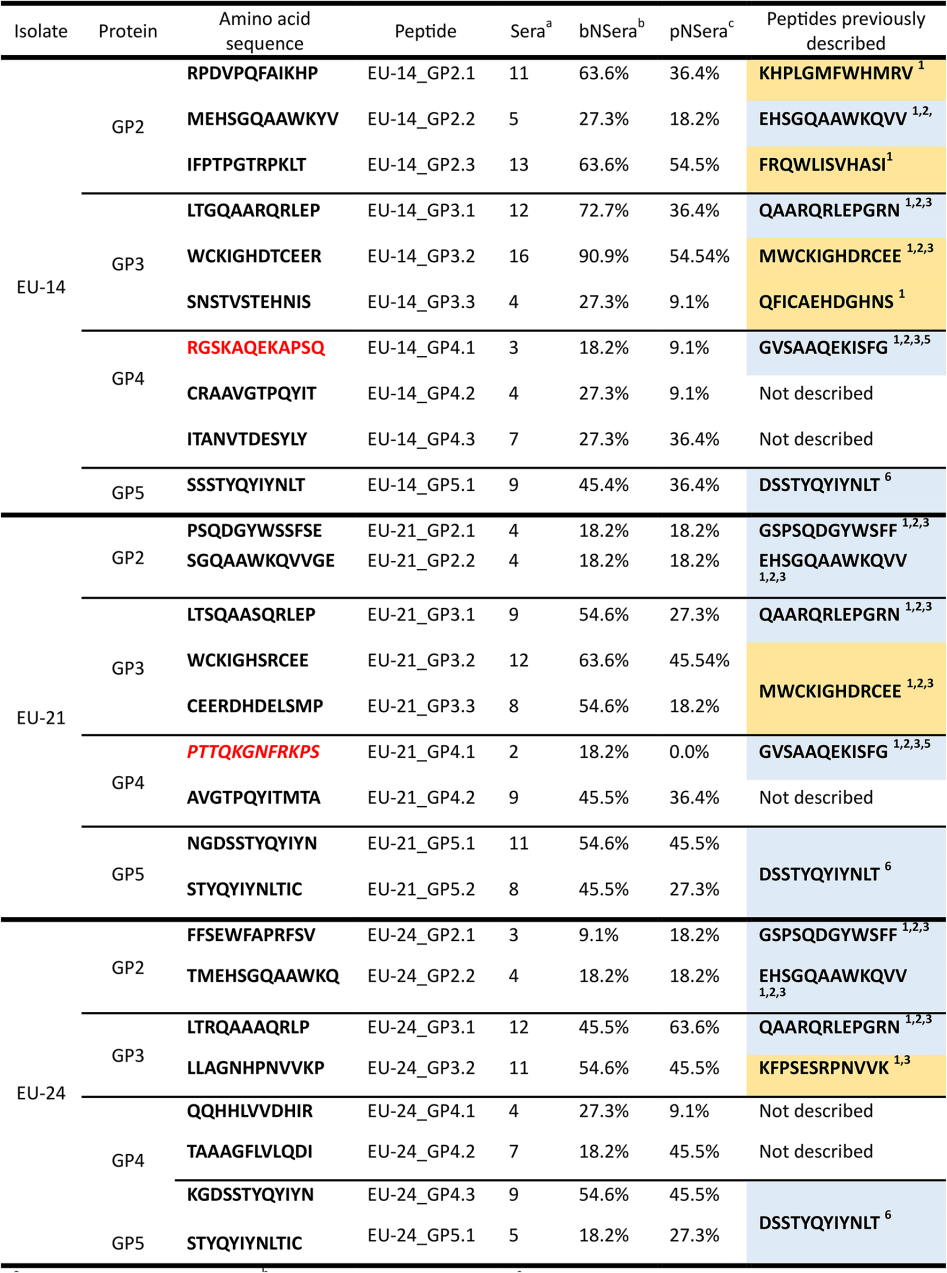
Peptides in the ectodomains of the envelope proteins of the isolates used in the study and their correspondence with peptides previously described in the literature for the Lelystad strain, prototype of PRRSV: only peptides recognized by at least 4 sera are shown, with the exception of peptides described as homologous NE of GP4. Neutralizing peptides are marked in blue and non-neutralizing peptides in orange. Peptides highlighted in red indicate those described as homologous NE of GP4

^a^: Number of reactive sera, ^b^: Broadly neutralizing sera; ^c^: Poor neutralizing sera

^1^: Vanhee et al. (2011); ^2^: de Lima et al. (2006); ^3^ : Oleksiewicz et al. (2000); ^4^: Meulenberg et al. (1997); ^5^: Costers et al. (2010); ^6^: Plagemann, (2004)

### Competition assays for the cellular receptor

**Fig. 1:**
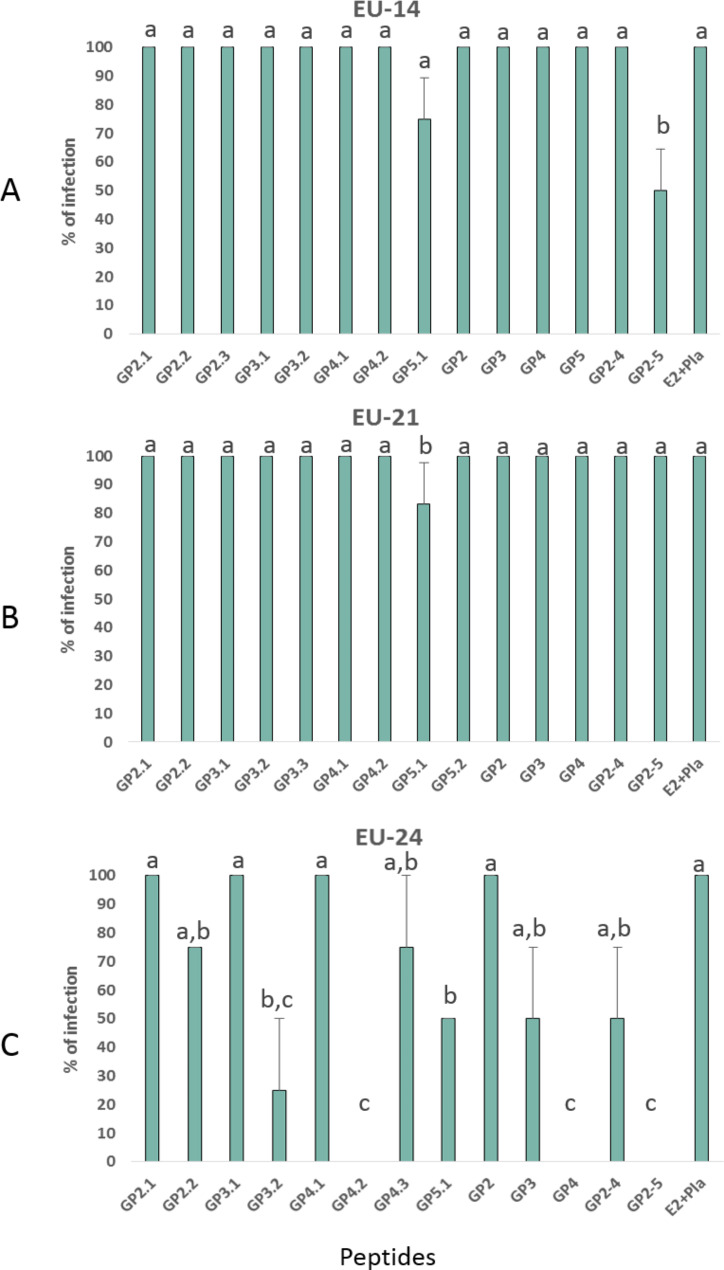
Competition assays for cellular receptors. Mean and standard deviation of the percentage of infected cells. Mix of irrelevant peptides, i.e. E2 (CSFV) and Pleurocidin (*P. americanus*) was included as control. All assays were conducted in duplicate and repeated three times. Different letters indicate statistical significance (*p* < 0.05)

The results of the competition assays for the cellular receptor showed that neither the negative control (i.e. DMEM) nor the diluents used to prepare the peptides (i.e. DMSO and PBS) had any toxic effect on the cell layer nor did they interfere with the viral infection at any concentration. Likewise, the presence of irrelevant peptides had no effect on the infectivity of the virus in any of the isolates studied. As it can be seen in Fig. 1, competition tests for the receptors of EU-14 and EU-21 showed that the peptides have a very limited ability to inhibit infectivity. Thus, in the case of the EU-14 isolate, the EU-14_GP5 (SSSTYQYIYNLT) peptide was able to inhibit 25% of the infectivity. This percentage increased to 50% inhibition when this peptide was combined with the rest of the selected peptides of GP2 to GP5 (Fig. 1A). In the case of the EU-21 isolate, the inhibition effect was even lower and only the use of the peptide EU-21_GP5.2 (STYQYIYNLTIC) produced a slight reduction in infectivity (Fig. 1B).

On the contrary, the peptides of the EU-24 isolate selected inhibited viral infection in a variable percentage, depending on the peptide analyzed (Fig. 1C). Thus, the peptides EU-24_GP2.2 (TMEHSGQAAWKQ), EU-24_GP4.3 (QQHHLVVDHIRL) and EU-24_GP3 inhibited viral infection by 25%; the peptides EU-24_GP5.1 (NGDSSTYQYIYN) and EU-24_GP2.4 inhibited viral infection by 50%, and the peptide EU-24_GP3.2 (WCKIGHSRCEE) inhibited up to 75% of viral infectivity. On the other hand, it is notable that the EU-24_GP4.2 peptide (TAAAGFLVLQDI), both individually and in combination with the rest of the GP4 peptides, was capable of completely inhibiting infectivity. Finally, the combination of all the selected peptides also completely inhibited viral infection.

### Seroneutralization inhibition assays

The results of the SN inhibition assays after incubation of the hyperimmune sera with the selected peptides are shown in Tables 4, 5 and 6. No statistically significant differences were observed among the peptides analyzed.

In the case of EU-21 and EU-24, no decrease in the neutralizing activity of the hyperimmune sera was observed after incubation with any of the peptides used in the experiment. When the assay was carried out with EU-14 isolate, peptides GP2.3, GP4.1 and GP2.5 and the mix of GP2, GP3 and GP4 were able to reduce 1 log2 in the NAs titer of homologous sera (Table 4). Likewise, the neutralizing activity of the heterologous hyperimmune sera against EU-14 isolate did not markedly decrease when they were incubated with any of the selected peptides, and only a small effect was observed with peptide GP4.1 and mix peptides GP2, GP3 and GP2-4.

**Table 4:** Titer of neutralizing antibodies (log_2_) against the EU-14 isolate in the presence of the selected peptides

Serum	GP2.1	GP2.2	GP2.3	GP3.1	GP3.2	GP4.1	GP4.2	GP5.1	GP2	GP3	GP4	GP5	GP2-4	GP2-5	E2 + Pl^a^
EU-14-1	7.0	6.0	5.0	6.0	6.0	6.0	6.0	6.0	5.0	5.0	5.0	6.0	7.0	6.0	6.0
EU-14-2	8.0	8.0	8.0	8.0	8.0	8.0	7.0	8.0	8.0	8.0	8.0	8.0	7.0	8.0	8.0
EU-14-3	8.0	8.0	8.0	8.0	8.0	8.0	8.0	8.0	8.0	8.0	8.0	8.0	8.0	8.0	8.0
EU-14-4	8.0	8.0	8.0	8.0	8.0	8.0	7.0	8.0	8.0	8.0	8.0	8.0	8.0	7.0	8.0
GMTh^b^	7.7	7.4	7.1	7.4	7.4	7.4	7.0	7.4	7.1	7.1	7.1	7.4	7.5	7.2	7.4
Sp-3-3	7.0	7.0	7.0	5.0	7.0	6.0	7.0	7.0	6.0	7.0	7.0	7.0	6.0	7.0	7.0
EU-21-4	6.0	6.0	6.0	6.0	6.0	6.0	6.0	6.0	5.0	5.0	6.0	6.0	6.0	6.0	6.0
GMT ht^c^	6.5	6.5	6.5	5.5	6.5	6.0	6.5	6.5	5.5	5.9	6.5	6.5	6.0	6.5	6.5

^a^: Mix of irrelevant peptides: E2 (CSFV) and Pleurocidin (*P. americanus*); b: GMT of NA against homologous sera. c: GMT of NA against heterologous sera. All neutralization-inhibition assays were conducted in duplicate and repeated three times

**Table 5:** Titer of neutralizing antibodies (log_2_) against the EU-21 isolate in the presence of the selected peptides

Serum	GP2.1	GP2.2	GP3.1	GP3.2	GP3.3	GP4.1	GP4.2	GP5.1	GP5.2	GP2	GP3	GP4	GP2-4	GP2-5	E2 + Pl^a^
EU-21-1	4.0	4.0	4.0	4.0	4.0	4.0	4.0	4.0	4.0	4.0	4.0	5.0	4.0	4.0	4.0
EU-21-2	4.0	4.0	4.0	4.0	4.0	4.0	4.0	5.0	5.0	4.0	3.0	5.0	5.0	5.0	5.0
EU-21-3	4.0	4.0	4.0	5.0	4.0	5.0	4.0	4.0	5.0	4.0	4.0	4.0	4.0	5.0	5.0
EU-21-4	4.0	4.0	5.0	4.0	4.0	4.0	4.0	4.0	4.0	5.0	5.0	5.0	5.0	4.0	5.0
GMTh^b^	4.0	4.0	4.2	4.2	4.0	4.2	4.0	4.2	4.5	4.2	3.9	4.9	4.5	4.5	4.7
Sp-3-1	4.0	4.0	4.0	3.0	4.0	4.0	4.0	4.0	4.0	3.0	4.0	4.0	4.0	4.0	4.0
EU-14-3	4.0	5.0	4.0	4.0	4.0	4.0	4.0	4.0	4.0	4.0	5.0	4.0	4.0	4.0	5.0
GMT ht^c^	4.0	4.5	4.0	3.5	4.0	4.0	4.0	4.0	4.0	3.5	4.5	4.0	4.0	4.0	4.5

^a^: Mix of irrelevant peptides: E2 (CSFV) and Pleurocidin (*P. americanus*); b: GMT of NA against homologous sera. c: GMT of NA against heterologous sera. All neutralization-inhibition assays were conducted in duplicate and repeated three times

**Table 6:** Titer of neutralizing antibodies (log_2_) against the EU-24 isolate in the presence of the selected peptides

Serum	GP2.1	GP2.2	GP3.1	GP3.2	GP4.1	GP4.2	GP4.3	GP5.1	GP2	GP3	GP4	GP2-4	GP2-5	E2 + Pl^a^
EU-24-1	7.0	8.0	8.0	7.0	7.0	8.0	8.0	7.0	7.0	8.0	7.0	7.0	8.0	7.0
EU-24-2	7.0	7.0	8.0	8.0	7.0	8.0	7.0	8.0	7.0	7.0	7.0	8.0	8.0	8.0
EU-24-3	7.0	8.0	8.0	8.0	8.0	8.0	7.0	7.0	7.0	7.0	7.0	8.0	8.0	8.0
EU-24-4	7.0	8.0	7.0	8.0	8.0	7.0	8.0	7.0	7.0	7.0	7.0	8.0	8.0	8.0
GMTh^b^	7.0	7.7	7.7	7.7	7.5	7.7	7.5	7.2	7.0	7.3	7.0	7.7	8.0	7.7
Sp-3-1	6.0	6.0	6.0	6.0	6.0	6.0	6.0	6.0	6.0	6.0	6.0	7.0	6.0	6.0
EU-14-3	5.0	5.0	5.0	5.0	5.0	5.0	6.0	6.0	5.0	5.0	5.0	5.0	5.0	5.0
GMT ht^c^	5.5	5.5	5.5	5.5	5.5	5.5	6.0	6.0	5.0	5.5	5.5	5.9	5.5	5.5

^a^: Mix of irrelevant peptides, E2 (CSFV) and Pleurocidin (*P. americanus*); b: GMT of NA against homologous sera. c: GMT of NA against heterologous sera. All neutralization-inhibition assays were conducted in duplicate and repeated three times

## Discussion

The investigation into the specificity of the antibody response upon PRRSV infection, with a focus on identifying both NEs and non-NEs, holds significant importance not only for the improving of knowledge on PRRSV immunology but also for it practical applications. Firstly, the identification of NE relevant for protection would facilitate the development of rapid diagnostic tests with high sensitivity and specificity, enabling the prompt and automated assessment of NA responses. Additionally, the identification of relevant epitopes would encourage the development of next-generation vaccines with improved formulations that incorporate these epitopes to enhance the efficacy of existing PRRSV vaccines. Consequently, numerous studies have been carried out with the objective of pinpointing biologically relevant epitopes [14–16, 18, 21, 22, 32].

The outcomes of these investigations suggest that NEs, which could be potentially crucial for robust protection against reinfections, are located in GP2, GP3, GP4, GP5, and M proteins [14]. Furthermore, in vivo studies utilizing chimeric viruses containing mosaic envelope protein sequences from various isolates identified the collaborative role of these proteins in fostering broadly NAs and in improving cross-protection [33].

Based on the existing knowledge, our study has focused on the analysis of the envelope proteins GP2, GP3, GP4, GP5 and M, due to their significance in the neutralizing antibody response, with the objective of identifying NEs, and more specifically linear NEs, that could be conserved and relevant for the induction of broadly reactive NA. To accomplish this aim we employed a panel of PRRSV isolates characterized on the basis of their susceptibility or resistance to neutralization and a collection of hyperimmune monospecific sera encompassing both, broadly reactive sera and sera with poor cross-reactivity against heterologous viruses in SN assays. Specifically, the use of sera with known cross-neutralization capacity is pivotal for distinguishing conserved NEs responsible for neutralizing heterologous isolates from those involved solely in neutralizing the immunization (i.e. homologous) virus.

The immunodominant peptides of various PRRSV envelope proteins have been successfully identified using the Pepscan technique [14]. Different systems have been proposed in the literature for the analysis of Pepscan signals. In this study, we have used the system established for PRRSV by Vanhee et al. [14] in which the signal of a particular peptide is related to that obtained for the set of peptides of the protein studied. The main advantage of this system is that it ensures that the signals identified are very specific. However, and with the objective of further confirming the specificity of the reactivity identified, the signals were evaluated using two other systems widely used for the analysis of the reactivity of different sera against specific peptides in Pepscan systems. The first one consists of the establishment of a cut-off point that corresponds to the average value of the optical density obtained with the negative sera plus two times the standard deviation [34]. The second one consists of qualifying as specific the signals whose value is at least three times higher than the average value of the negative controls [35]. The results obtained in our study using these systems are very similar to those obtained using the method proposed by Vanhee et al. [14] (data not shown). The consistency of the results across different methods suggests the reliability of Pepscan for consistent and reliable outcomes and supports the immunogenic potential of the peptides identified.

The results of our study reveal that the linear peptides of the ectodomains of the PRRSV-1 main envelope proteins exhibit a relatively low immunogenicity, as evidenced by the relatively low number of reactive peptides and, more importantly, by the low number of sera that typically recognize the reactive peptides. Thus, most synthetic peptides were recognized by only one or two sera, regardless the protein or the protein region considered and none of them was recognized by all sera. This observed lack of broad reactivity has been previously described in the literature [14, 36] and challenges the antigenic relevance of these areas. The reasons for this low recognition rate have not been identified but they can be diverse. On one hand, it is possible that these regions are poorly exposed to the immune system as many of the studied proteins are minor virion proteins and their relative expression in the infected cell is low, compared to other immunodominant proteins, such as the N protein or nsp-7, which induce a high antibody response in infected individuals [37–39]. These differences in immune exposure might motivate the development of a weaker immune response to these antigenic determinants. However, although it might be the case for proteins poorly recognized by convalescent pigs, such as the minor envelope glycoproteins GP2, GP3 and GP4 [40], it is unlikely to be the case for the GP5 or M proteins, which are generally well recognized by infected pigs [41]. An alternative explanation is that the antigenic variability of PRRSV makes the recognition of heterologous strains quite difficult, to the point that hyperimmune monospecific sera might not be able to recognize some antigenic determinant present in heterologous strains. In this line of thinking, the lack of cross-reactivity in serological studies carried out using polyclonal and monoclonal antibodies has been repeatedly demonstrated in the literature and constitutes a hallmark of PRRSV [23, 42–45]. Finally, it should be kept in mind that the capacity for specific epitope recognition is genetically determined by the individual’s B-cell repertoire [46]. The results of our study indicate that the reactive peptides are not generally recognized, not even by all homologous sera. Thus, it is likely that the poor recognition of the reactive linear peptides identified is the result of the combination of the PRRSV variability, that might prevent the recognition of antigenic determinants of heterologous viruses, and the individual variability in B cell repertoires, that may lead to non-recognition by even most individuals within a population.

Despite the overall low immunogenicity of the peptides studied, certain regions with greater immunogenicity have been identified in some of the protein studied. Among them, an AR identified in the ectodomain of GP3 stands out for the high number of sera of different specificities that recognize it in all three viruses analyzed. These results are in agreement with the results of previous studies in which the same region has been widely recognized by convalescent sera in both PRRSV-1 and PRRSV-2 [14, 34, 36]. Remarkably, a NE has been described within this AR for PRRSV-1 [14]. As the amino acidic sequence of this region is fairly well conserved between PRRSV isolates and recognition by sera of different specificity has been systematically described, it could be speculated that this epitope is relevant for cross-neutralization. Nevertheless, the data obtained in this study do not suggest a crucial involvement of this epitope in the effective neutralization of heterologous strains, as this region is recognized by broadly reactive sera and by sera with low cross-reactivity in SN assays. Finally, it is noteworthy to mention that another AR, previously described within PRRSV-1 and PRRSV-2 GP3 [14, 34, 36], has been identified in our study. However, the main epitope in this region has been classified as non-neutralizing by others [12, 14] and despite it recognition by a significant number of sera of different specificities, its significance in protection seems to be inconsequential.

The results of our study confirm that the ectodomain of GP5 also harbors a widely accepted and fairly conserved AR, that is recognized by both homologous and heterologous sera with varying degrees of cross-reactivity. This particular region has been deemed crucial for protection, given the identification of one of the first NEs in this region [19]. Later, the role of this epitope as NE has been questioned for PRRSV-1 isolates [14] and even for PRRSV-2 [47]. However, recent studies utilizing machine learning techniques has revealed that specific modifications in the GP5 NE sequence could significantly alter the antigenic distance, measured by SN assays, between PRRSV-1 isolates, suggesting a potential role for this epitope as a NE, despite previous uncertainties [27].The results of our study indicate that, shall the region contain a NE, it is not preeminently involved in broad-spectrum neutralization, as the proportion of broadly-reactive and poorly-reactive sera that react with these peptides is similar.

In the case of GP4 ectodomain, a highly immunogenic NE has been described in PRRSV-1 isolates [32]. Consistently, in our study, all homologous sera recognized the corresponding peptides in EU-14 and EU-21 isolates, irrespectively of their cross-reactivity. On the contrary, no recognition by heterologous sera was recorded. This finding was not unexpected as the most outstanding characteristic of this epitope is its high variability, which may contribute to the nearly unique sequence observed in each virus isolate studied [23], potentially explaining the limited cross-reactivity exhibited by the viruses in most cases. This variability might be an escape mechanism of PRRSV as it allows the virus to undergo evolution during in vivo infection as animals develop specific NA [48]. This adaptive process leads to the creation of mutants resistant to neutralization [32] that have the potential to continue circulating in immune populations.

An unexpected observation was the lack of recognition of the GP4 NE of the EU-24 isolate, not even by any of the homologous sera used in the study. Although this epitope seems to be present in all PRRSV-1 virus isolates studied so far, it has not been documented in PRRSV-2 isolates [34]. It is plausible that certain PRRSV-1 isolates, particularly those more diverse and distant from the LV, the PRRSV-1 prototype strain, such as the Italian strain used in this study, may lack this epitope. This absence could lead to the adaptative immune response of infected animals targeting alternative epitopes. In fact, a peptide located upstream of the typical NE of GP4, and previously undescribed for PRRSV-1, has been identified in EU-24 by a variety of sera, both homologous and heterologous. The biological relevance of this peptide and its existence in alternative viral isolates deserves further investigation.

Finally, even though a NE and other immunodominant regions have been previously described in GP2 [14, 34, 36], linear peptides from this protein were poorly recognized by the hyperimmune sera used in this study. These results are consistent with those obtained by others [14] and confirm the very poor immunogenicity of this protein, despite its biological relevance [11].

On the other hand, it should be mentioned that some sera were able to recognize several peptides from proteins of different virus isolates. Specifically, one of the sera against the EU-9 isolate showed a very broad reactivity, recognizing a high number of peptides. This phenomenon, which has been previously described [24], could be due to non-specific reactions, so additional studies would have to be carried out to verify the specificity of the reaction.

The Pepscan results were used to make a selection of peptides, targeting the most immunogenic ARs in each envelope protein and virus. In addition, peptides previously identified as NE were also selected, regardless of their recognition rate. All these peptides were used to further study the corresponding AR and determine their role in virus neutralization. To do so, two different approaches were followed. In the first place, a competition assay was carried out between the selected linear peptides and the virus for binding to cellular receptors. If the peptides bind to cellular receptors, mimicking virus binding, they will compete with the virus and a reduction in virus infectivity will be observed [31]. However, the results obtained in our study indicate that the addition of either individual peptides or combinations of peptides to the cell culture before infection with two of the virus isolates used was not sufficient to block infection. These results are in agreement with those obtained by Robinson et al. [31] using a PRRSV-2 isolate and different peptide concentrations. However, and surprisingly, some peptides or combinations of peptides from the third isolate used in our study (i.e. EU-24) were able to partially or even completely block infectivity. Specifically, the addition of a GP3 peptide led to a very marked inhibition of viral infectivity and incubation with one of the GP4 peptides or with a mix of GP2 to GP5 peptides completely inhibited virus replication, indicating that these peptides might play a significant role in virus-cell interaction. Although the reasons for these discrepancies have not been elucidated, our results seem to indicate that different PRRSV isolates might differ in their interactions with cell receptors. In this line of thinking, some differences have been observed between PRRSV-1 and PRRSV-2 isolates in their interaction with CD163 [49] and also in the range of susceptible cell subpopulations depending on PRRSV pathogenicity [50].

Finally, the selected peptides were incubated with the hyperimmune sera with the objective of blocking the specific antibodies against those peptides, following previously established protocols [14, 31]. If the peptides were the target of the NAs, they would compete with the virus in binding to them, thus inhibiting their neutralizing activity and increasing the infectivity of the virus in SN assays [31]. However, the results obtained indicate that the peptides used do not have any effect on the neutralizing capacity of the selected hyperimmune sera. These results are in line with those obtained by Robinson et al. [31] using a PRRSV-2 isolate, but contrast with the observations made by Vanhee et al. [14]. These authors demonstrated that antibodies directed against some of the peptides in the ARs identified in GP2, GP3 and GP4 have neutralizing activity. Although the results of both studies seem to be contradictory, they can be explained by the different methodological approaches followed. Thus, Vanhee et al. [14] purified the antibodies specific to the assayed peptides by affinity column and revealed that these purified antibodies had neutralizing activity in SN assays. This fine experimental design allows demonstrating that those epitopes can elicit NAs but does not exclude the presence or the role of NAs of different specificities in PRRSV immune sera. In our study, and in the study carried out by Robinson et al. [31], polyclonal hyperimmune sera were incubated with selected peptides. In this experimental approach, it is possible that the concentration of NAs specific for the assayed peptides was lower than in the study carried out by Vanhee et al. [14], preventing their detection. Besides, in polyclonal sera the presence of other antibodies, of different specificities, might have produced steric hindrance or another type of impediment in the binding to the NE studied abrogating their biological effect. However, another possible, and more likely, explanation is that, although NAs specific for the peptides tested have been blocked, other PRRSV-specific NAs present in the sera, directed against other NEs, either linear or, more likely, conformational, might have acted blocking the virus infectivity and concealing the presence of NAs against the peptides tested in this study. In this line of thinking, it should be kept in mind that the minor envelope proteins are folded and interact during virus assembly [11] and thus, the conformational nature of the peptides in the native protein might be different and non-contiguous or conformation-dependent epitopes might span different regions or even proteins. Finally, the cell type used in the SN assays might have influenced the results. Thus, in our study, and in the study carried out by Robinson et al. [31], SN assays were carried out in MARC-145, a cell line highly permissive to PRRSV infection [51] that contains the monkey CD163, instead of swine, and does not contain Siglec-1 but other Siglecs [52] while Vanhee et al. [14] used porcine alveolar macrophages. The use of MARC-145 cells for PRRSV functional analysis may be controversial. Firstly, PRRSV often requires adaptation, both at non-structural and structural proteins, especially at GP2-GP3, to replicate in MARC-145 cells [53, 54]. This adaptation may alter the virus-host cell interaction and may not accurately represent what occurs in vivo infections. Additionally, the interaction between PRRSV-1 and MARC-145 cells is not well understood; although initial binding is facilitated by heparan sulfate, the mechanisms of internalization remain unclear. One possibility is that PRRSV-1 may enter MARC-145 cells through spontaneous internalization, a process described in other viruses like rotaviruses and HIV [55, 56], which have limited host cell specificity but can still grow in stable cell lines. This spontaneous internalization might bypass critical receptor-mediated steps that are essential in natural PRRSV infections of macrophages. Finally, using MARC-145 grown viruses for functional analysis may introduce biases. Antibodies developed against viruses grown in MARC-145 or other non-macrophage cells may not effectively bind viral structures as they appear in the natural host, further compromising the relevance of such assays. For these reasons, while MARC-145 cells offer a convenient experimental platform, they present certain limitations for PRRSV functional analysis. Future studies could be carrying in porcine macrophages to confirm the validity of the results of this study.

## Conclusions

The results of our study confirm that the ARs described for PRRSV are relatively well conserved across PRRSV isolates, although some of them might present certain particularities, as it is the case for the Italian isolate included in our study. However, the antigenicity of those ARs seems to be limited as the number of individuals that recognize each peptide is usually low. Besides, there are no clear differences in the peptide recognition pattern between hyperimmune sera of broad and low cross-reactivity in SN assays, which might indicate that broadly reactive NAs are not directed against NEs present in any of the linear peptides studied. Thus, even though the results of this study do not allow excluding the possibility of linear epitopes contributions to virus neutralization, the cross-reactive NA response to these epitopes is probably of minor importance. In the light of these results, it could be speculated that the broad cross-reactivity exhibited in SN assays by the sera used in our study might be the consequence of the response to critical and conserved conformational epitopes or, alternatively, the result of the combined response to a variety of different epitopes none of which would be essential.

## Electronic supplementary material

Below is the link to the electronic supplementary material.

Supplementary Material 1

## Data Availability

No datasets were generated or analysed during the current study.

## Abbreviations

PRRS: Porcine reproductive and respiratory syndrome
PRRSV: Porcine reproductive and respiratory syndrome virus
ORF: Open reading frame
kb: kilo-base-pair
mRNA: messenger ribonucleic acid
NAs: neutralizing antibodies
ARs: Antigenic regions
NE: Neutralizing epitope
DMEM: Dulbecco’s Modified Eagle Medium
OD: optical density
CPE: cytopathic effect
FBS: Fetal Bovine Serum
PBS: phosphate buffer saline
TCID50: Median tissue culture infectious dose
SN: seroneutralization assay
DMSO: Dimethyl sulfoxide
CSFV: Classical swine fever virus
