# Supplementary material for: Linear epitopes of PRRSV-1 envelope proteins ectodomains are not correlated with broad neutralization
Source: Porcine Health Manag. 2024 Oct 21;10:44. doi: 10.1186/s40813-024-00393-7 (PMC11492654; doi:10.1186/s40813-024-00393-7)
Supplement: Supplementary file 1 — Supplementary Material 1 [file 40813_2024_393_MOESM1_ESM.docx]

**Supplementary data 3.1.** Peptides in the ectodomains of the GP2 and GP3 of the EU-14 isolate used in the study and their correspondence with peptides previously described in the literature for the Lelystad strain, prototype of PRRSV-1. Neutralizing peptides are marked in blue and non neutralizing peptides in orange.

| Protein | Amino acid sequence | bSera^a^ | pSera^b^ | Peptides previously described |
| --- | --- | --- | --- | --- |
| GP2 | **^32^LPYCLGSPLQGG^43^** | 1 (Sp-3)^c^ | 1 (Sp-3) | **GSPSQDGYWSFF^b^ ^1,2,3^** |
|  | **^56^FSVRALPFTLPN^67^** | 1 (EU-9) | 1 (EU-9) | No previously described |
|  | **^72^YEGLLPNCRPDV^83^** | 1 (Sp-28) | 1 (Sp-3) | No described |
|  | **^76^LPNCRPDVPQFA^87^** | 2 (EU-14) | 3 (Sp-2/Sp-28/EU-9) | Not described |
|  | **^80^RPDVPQFAIKHP^91^** | 7 (Sp-2, EU-21, EU-9, EU-14, Sp-5, Sp-28) | 4 (Sp-2/Sp-3/EU-9/EU-14) | **KHPLGMFWHMRV** ^1^ |
|  | **^104^IDEMVSRRIYQT^115^** | 0 | 1 (Sp-2) | Not described |
|  | **^116^MEHSGQAAWKYV^127^** | 3 (Sp-3, Sp-28, EU-14) | 2 (EU-9, Sp-5) | **EHSGQAAWKQVV ^1,2,3^** |
|  | **^121^GQAAWKYVGEAT^132^** | 3 (EU-9/Sp-2/Sp-3) | 0 |  |
|  | **^124^WKYVVGEATLTK^135^** | 3 (EU-9/Sp-3/EU-21) | 1 (EU-9) |  |
|  | **^128^VGEATLTKLSTL^139^** | 3 (EU-9/Sp-3/EU-21) | 0 |  |
|  | **^152^EADSCRFLSSRL^163^** | 1 (EU-9) | 1 (EU-11) | Not described |
|  | **^170^SSRLVMLKNLAV^171^** | 0 | 1 (EU-9) |  |
|  | **^176^LQYNTTLDRVEL^187^** | 1 (EU-14) | 3 (Sp-2, EU, 14,EU-23) | **FRQWLISVHASI^1^** |
|  | **^180^TTLDRVELIFPT^191^** | 3 (Sp-3/EU-9/EU-21) | 0 |  |
|  | **^188^IFPTPGTRPKLT^199^** | 7 (Sp-3/Sp-5/EU-9/EU-21/EU-14) | 6 (Sp-2/EU-9/EU-14/EU-23/EU-24) |  |
|  | **^196^PKLTNFRQWLIG^207^** | 2 (Sp-3/EU-9) | 0 |  |
| GP3 | **^54^CKPCLTGQAARQ^65^** | 2 (EU-14) | 2 (EU-14) | **QAARQRLEPGRN ^1,2,3^** |
|  | **^58^LTGQAARQRLEP^69^** | 8 (Sp-2, Sp-3,Sp-5, EU-14, EU-9, EU-21, ) | 4 (Sp-2/EU-14, EU-9) |  |
|  | **^62^AARQRLEPGRNM^73^** | 7 (Sp-2, Sp-3, Sp-5, Sp-28, EU-9, EU-21, EU-14) | 4 (Sp-2/EU-9/EU-11/EU-24) |  |
|  | **^66^RLEPGRNMWCKI^77^** | 1 (EU-9) | 0 |  |
|  | **^74^WCKIGHDTCEER^85^** | 10 (Sp-2/Sp-3/Sp-5/EU-9/EU-21/EU-23/EU-24, EU-14) | 6 (Sp-2/Sp-3/EU-14, EU-23/EU-24) | **MWCKIGHDRCEE ^1,2,3^** |
|  | **^82^CEERDHDELSMS^93^** | 8 (Sp-2/Sp-3/Sp-5/Sp-28/EU-9/EU-21)Eu-14) | 3 (Sp-2/Sp-5/EU-9) | **RDHDELLMSIPS^1,2^** |
|  | **^86^DHDELSMSIPSG^97^** | 2 (EU-9/EU-21) | 0 |  |
|  | **^90^LSMSIPSGYDNL^101^** | 1 (EU-9) | 0 | **GYDNLKLEGYYA ^1,7^** |
|  | **^94^IPSGYDNLKLEG^105^** | 2 (Sp-3/EU-9) | 0 |  |
|  | **^150^SNSTVSTEHNIS^161^** | 3 (Sp-5, EU-9, EU-21) | 1 (EU-14) | **QFICAEHDGHNS ^1^** |
|  | **^110^LAFLSFSYAAQF^121^** | 1 (EU-14) | 0 | **GYYAWLAFLSFS ^7^** |
|  | **^170^HQVDGGNWFHLE^181^** | 1 (EU-9) | 0 | **EWLRPLFSSWLV ^1^** |
|  | **^122^LPVSWSFRTSIA^233^** | 0 | 1 (EU-11) | Not described |

a: Broadly neutralizing sera; b: Poor cross-neutralizing sera; c: Isolate use for immunization

1: Vanhee et al. (2011); 2: de Lima et al. (2006); 3 : Oleksiewicz et al. (2000); 4: Meulenberg et al. (1997); 5: Costers et al. (2010); 6: Plagemann, (2004); 7: Zhou et al. (2007)

**Supplementary data 3.2.** Peptides in the ectodomains of the GP2 and GP3 of the EU-14 isolate used in the study and their correspondence with peptides previously described in the literature for the Lelystad strain, prototype of PRRSV-1. Neutralizing peptides are marked in blue and non neutralizing peptides in orange.

| Protein | Amino acid sequence | bSera^a^ | pSera^b^ | Peptides previously described |  |
| --- | --- | --- | --- | --- | --- |
| GP4 | **^44^FMVLQNINCLQS^55^** | 0 | 1 (Sp-2)^c^ | Not described |  |
|  | **^48^QNINCLQSRGSK^59^** | 1 (EU-14) | 0 | Not described |  |
|  | **^52^CLQSRGSKAQEK^63^** | 2 (EU-14) | 1 (EU-14) | **GVSAAQEKISFG ^1,2,3,5^** |  |
|  | **^56^RGSKAQEKAPSQ^67^** | 2 (EU-14) | 1 (EU-14) |  |  |
|  | **^68^CRAAVGTPQYIT^79^** | 3 (Sp-3/EU-21) | 0 | Not described |  |
|  | **^72^VGTPQYITITAN^83^** | 1 (Sp-3) | 0 | Not described |  |
|  | **^76^QYITITANVTDE^87^** | 1 (EU-9) | 0 | Not described |  |
|  | **^80^ITANVTDESYLY^91^** | 3 (Sp-5, EU-21) | 4 (Sp-2/Sp-3/EU-11/ EU-23) | Not described |  |
|  | **^84^VTDESYLYNADL^95^** | 4 (Sp-5, EU-9, EU-21) | 2 (Sp-3/EU-23) | Not described |  |
|  | **^108^MSEKGFKVIFGN^119^** | 1 (EU-9) |  | Not described |  |
|  | **^116^IFGNVSGVVSAC^127^** | 3 (Sp-5, EU-9) | 1 (Sp-2) | Not described |  |
|  | **^128^VNFTDYVAHVTQ^139^** | 0 | 1 (EU-11) | Not described |  |
| GP5 | **^33^DGNDSSSTYQYI^44^** | 2 (Sp-2/Sp-5) | 1 (EU-24) | **DSSTYQYIYNLT ^6^** |  |
|  | **^37^SSSTYQYIYNLT^48^** | 5 (Sp-2, Sp-5, EU-9,EU-14) | 4 (Sp-5/EU-14/EU-24) |  |  |
|  | **^41^YQYIYNLTICEL^52^** | 2 (Sp-28, EU-14) | 1 (EU-14) |  |  |
|  | **^85^TTSHFFDALGLG^96^** | 1 (Sp-28) | 2 (Sp-2/EU-14) | Not described |  |
|  | **^93^LGLGAVSITGFF^104^** | 1 (EU-9) | 0 | Not described |  |
|  | \| **^157^WKSPIVVEKLGK^168^** \| \| --- \| \| **^165^KLGKAEVGSDLV^175^** \| | 0 | 2 (Sp-2/EU-9) | **IVVEKLGKAEVD^1^** | |
|  |  | 1 (EU-14) | 0 | **KLGKAEVDGNLV^1,7^** | |
| M | **^1^MGGLDNFCYDPT^12^** | 0 | 1 (EU-9) | **MGGLDDFCNDPI ^1^** | |

a: Broadly neutralizing sera; b: Poor cross-neutralizing sera; c: Isolate use for immunization

1: Vanhee et al. (2011); 2: de Lima et al. (2006); 3 : Oleksiewicz et al. (2000); 4: Meulenberg et al. (1997); 5: Costers et al. (2010); 6: Plagemann, (2004); 7: Zhou et al. (2007)

**Supplementary data 1.1.** Peptides in the ectodomains of the GP2 and GP3 of the EU-21 isolate used in the study and their correspondence with peptides previously described in the literature for the Lelystad strain, prototype of PRRSV-1. Neutralizing peptides are marked in blue and non neutralizing peptides in orange.

| Protein | Amino acid sequence | bSera^a^ | pSera^b^ | Peptides previosuly described |
| --- | --- | --- | --- | --- |
| GP2 | **^31^SLPYCLGSPSQD^42^** | 1(Sp-28)^c^ | 1 (EU-9) |  |
|  | **^35^CLGSPSQDGYWS^46^** | 2 (Sp-28/EU-21) | 1 (EU-9) | **GSPSQDGYWSFF ^1,2,3^** |
|  | **^39^PSQDGYWSSFSE^50^** | 2 (Sp-28/EU-21) | 2 (EU-9/EU-23) |  |
|  | **^43^GYWSSFSEWFAP^54^** | 2 (Sp-28/EU-21) | 1 (EU-23) |  |
|  | **^79^CRPDVPQFAFKH^90^** | 1 (EU-14) | 1 (EU-14) |  |
|  | **^95^LWHMRVSHLIDE^106^** | 1 (EU-9) |  | KHPLGMFWHMRV^1^ |
|  | **^111^RIYQTMEHSGQA^122^** | 1 (EU-9) | 2 (EU-11/EU-23) |  |
|  | **^115^TMEHSGQAAWKQ^126^** | 1 (Sp-3) | 2 (EU-9/Sp-5) |  |
|  | **^119^SGQAAWKQVVGE^130^** | 2 (Sp-3/EU-9) | 2 (EU-9/Sp-5) | **EHSGQAAWKQVV ^1,2,3^** |
|  | **^123^AWKQVVGEATLT^134^** | 1 (EU-9) | 1 (EU-9) |  |
|  | **^127^VVGEATLTKLSR^138^** | 1 (EU-9) | 1 (EU-9) |  |
|  | **^143^THFQYLAAVEAD^154^** | 1 (EU-9) |  | Not described |
|  | **^147^YLAAVEADSCRF^158^** | 1 (EU-9) | 2 (EU-11/Sp-5) |  |
|  | **^151^VEADSCRFLSSR^162^** | 2 (Sp-3/EU-9) | 1 (EU-11) |  |
|  | **^155^SCRFLSSRLVML^166^** | 1 (Sp-3) | 1 (EU-11) |  |
|  | **^171^VGNVSLQYNTTL^182^** | 1 (Sp-3) |  |  |
|  | **^175^SLQYNTTLDRVE^186^** | 1 (EU-14) |  | FRQWLISVHASI ^1^ |
|  | **^179^TDFRQWLISVHA^190^** | 1 (EU-9) |  |  |
| GP3 | **^58^LTSQAASQRLEP^69^** | 6  (Sp-2/Sp-3/EU-14/Sp-5) | 3 (Sp-2/EU-23) | **QAARQRLEPGRN ^1,2,3^** |
|  | **^62^AASQRLEPGRNM^73^** | 5  (Sp-2/Sp-3/EU-21/Sp-5) | 3 (Sp-2/EU-24/  EU-23) |  |
|  | **^66^RLEPGRNMWCKI^77^** | 3 (EU-21/EU-9/Sp-28) | 3 (EU-9/EU-24) |  |
|  | **^74^WCKIGHSRCEE^85^** | 7  (Sp-2/Sp-3/EU-21/Sp-5) | 5 (Sp-2/Sp-3  /EU-24/Sp-5) | MWCKIGHDRCEE ^1,2,3^ |
|  | **^78^GHSRCEERDHD^89^** | 2 (EU-21/Sp-28) | 1 (EU-9) |  |
|  | **^82^CEERDHDELSMP^93^** | 6  (Sp-2/Sp-3/EU-21/EU-14/Sp-5) | 2 (Sp-2/EU-9) |  |
|  | **^86^DHDELSMPIPSG^97^** | 4 (EU-21/EU-9/Sp-28) | 1 (EU-9) | RDHDELLMSIPS ^1,2^ |
|  | **^90^LSMPIPSGYDNL^101^** | 1 (EU-21) |  |  |
|  | **^94^IPSGYDNLKLEG^105^** | 1 (EU-9) |  | GYDNLKLEGYYA ^1,7^ |
|  | **^110^LAFLSFSYAAQF^121^** | 1 (EU-14) |  | GYYAWLAFLSFS |
|  | **^146^EHDGQNSTISTG^157^** |  | 1 (EU-14) | QFICAEHDGHNS ^1^ |
|  | **^154^ISTGHNISASYA^165^** | 1 (EU-9) |  |  |
|  | **^174^GGNWFHLEWLRP^185^** | 1 (EU-9) |  | EWLRPLFSSWLV ^1^ |
|  | **^198^FLRRSPASPVSR^209^** | 1 (EU-21) |  | Not described |
|  | **^202^SPASPVSRRIYQ^213^** | 1 (EU-21) | 1 (EU-11) | Not described |
|  | **^222^LPVSWSFKTLVA^133^** | 1 (EU-9) |  | Not described |
|  | **^242^ISGSRPNVAKPS^253^** | 1 (EU-9) |  | KFPSESRPNVVK ^1,3^ |
|  | **^250^AKPSVPLNTSR^261^** | 1 (EU-9) |  |  |

a: Broadly neutralizing sera; b: Poor cross-neutralizing sera; c: Isolate used for immunization

1: Vanhee et al. (2011); 2: de Lima et al. (2006); 3 : Oleksiewicz et al. (2000); 4: Meulenberg et al. (1997); 5: Costers et al. (2010); 6: Plagemann, (2004); 7: Zhou et al. (2007)

**Supplementary data 1.2.** Peptides in the ectodomains of the GP4 and GP5 of the EU-21 isolate used in the study and their correspondence with peptides previously described in the literature for the Lelystad strain, prototype of PRRSV-1. Neutralizing peptides are marked in blue and non neutralizing peptides in orange.

| Protein | Amino acid sequence | bSera^a^ | pSera^b^ | Peptides previously described |
| --- | --- | --- | --- | --- |
| GP4 | **^23^CKPCFSTHLSDI^34^** | 1 (EU-9)^c^ |  | Not described |
|  | **^27^FSTHLSDIKTNT^38^** | 1 (EU-9) | 1 (Sp-2) | Not described |
|  | **^35^KTNTTAAAGFMV^46^** | 1 (EU-9) |  | Not described |
|  | **^47^GFMVLQDISCLS^58^** | 1 (EU-9) | 1 (EU-11) | Not described |
|  | **^51^SCLSPTTQKGNF^62^** | 2 (EU-21) |  | **GVSAAQEKISFG ^1,2,3,5^** |
|  | **^55^PTTQKGNFRKPS^66^** | 2 (EU-21) |  |  |
|  | **^59^KGNFRKPSQCRE^70^** | 2 (EU-21) |  |  |
|  | **^63^RKPSQCREAVGT^74^** | 2 (EU-21) |  |  |
|  | **^67^QCREAVGTPQYI^78^** | 3 (Sp-3/EU-21) | 1 (EU-24) | ISFGKSSQCREA ^1,2^ |
|  | **^71^AVGTPQYITMTA^82^** | 5 (Sp-3/EU-21/02V48/Sp-28) | 4 (Sp-2/EU-11/EU-23) | Not described |
|  | **^75^PQYITMTANVTD^86^** | 1 (Sp-3) |  | Not described |
|  | **^79^TMTANVTDESYL^90^** | 2 (EU-21/Sp-5) | 2 (Sp-3/249773) | Not described |
|  | **^83^NVTDESYLYNAD^94^** | 2 (EU-21/Sp-5) | 2 (Sp-3/249773) | Not described |
|  | **^91^YNADLLMLSACL^102^** | 1 (EU-9) |  | Not described |
|  | **^123^VVSACVNFTDYV^134^** | 2 (EU-9/EU-14) | 2 (Sp-2/EU-14/EU-11) | Not described |
| GP5 | **^31^FADGNGDSSTYQ^42^** | 1 (EU-9) |  | **DSSTYQYIYNLT ^6^** |
|  | **^35^NGDSSTYQYIYN^46^** | 6  (Sp-2/EU-9/EU-14/Sp-5/Sp-28) | 5  (Sp-3/EU-14/EU-24/Sp-5 |  |
|  | **^39^STYQYIYNLTIC^50^** | 5 (Sp-2/EU-9/EU-14/Sp-28) | 3 (EU-14/EU-24/Sp-5) |  |
|  | **^47^LTICELNGTNWL^58^** | 1 (EU-9) |  | Not described |
|  | **^67^ETFVLYPVATHI^78^** |  | 2 (EU-11) | Not described |
|  | **^71^LYPVATHILSLG^82^** | 1 (EU-9) |  |  |

a: Broadly neutralizing sera; b: Poor cross-neutralizing sera; c: Isolate use for immunization

1: Vanhee et al. (2011); 2: de Lima et al. (2006); 3 : Oleksiewicz et al. (2000); 4: Meulenberg et al. (1997); 5: Costers et al. (2010); 6: Plagemann, (2004); 7: Zhou et al. (2007)

**Supplementary data 2.1.** Peptides in the ectodomains of the GP2 and GP3 of the EU-24 isolate used in the study and their correspondence with peptides previously described in the literature for the Lelystad strain, prototype of PRRSV-1. Neutralizing peptides are marked in blue and non neutralizing peptides in orange.

| Protein | Secuencia aminoacídica | bSera^a^ | pSera^b^ | Peptides previously described |
| --- | --- | --- | --- | --- |
| GP2 | **^43^GYWSFFSEWFAP^54^** | 1(EU-21)^c^ | 1 (EU-23) |  |
|  | **^47^FFSEWFAPRFSV^58^** | 1 (EU-9) | 2 (EU-11) | **GSPSQDGYWSFF ^1,2,3^** |
|  | **^79^CRPDVPQFAIKH^90^** | 1 (EU-14) | 1 (EU-11) | KHPLGMFWHMRV ^1^ |
|  | **^87^AIKHPLGILWHM^98^** | 1 (EU-9) |  |  |
|  | **^95^LWHMRVSRLIDE^106^** | 1 (EU-9) |  |  |
|  | **^111^RVYQTMEHSGQA^122^** |  | 1 (Sp-5) |  |
|  | **^115^TMEHSGQAAWKQ^126^** | 2 (Sp-3/Sp-28) | 2 (EU-9/Sp-5) | **EHSGQAAWKQVV ^1,2,3^** |
|  | **^119^SGQAAWKQVVTE^130^** | 2 (Sp-3/EU-9) | 2 (EU-9/Sp-5) |  |
|  | **^131^ATLTKLSQLDIV^142^** | 1 (EU-9) | 2 (Sp-2/EU-11) |  |
|  | **^135^KLSQLDIVTHFQ^146^** | 1 (EU-9) | 2 (Sp-2/EU-11) |  |
|  | **^139^LDIVTHFQHLAA^150^** |  | 2 (Sp-2/EU-11) |  |
|  | **^147^HLAAVEADSCRF^158^** | 1 (Sp-2) |  |  |
|  | **^159^LSSRLVMLKNLA^170^** |  | 1 (Sp-2) |  |
|  | **^171^VGNVSLQYNTTL^182^** | 1 (Sp-3) |  |  |
|  | **^175^SLQYNTTLDRVE^186^** |  | 1 (EU-23) |  |
| GP3 | **^50^NYTICEPCLTRQ^61^** |  | 1 (EU-24) | ***QAARQRLEPGRN ^1,2,3^*** |
|  | **^54^CEPCLTRQAAAQ^65^** |  | 1 (EU-24) |  |
|  | **^58^LTRQAAAQRLEP^69^** | 5 (Sp-2/Sp-3/Sp-5) | 7 (Sp-2/Sp-3/EU-14/EU-24/EU-23 |  |
|  | **^62^AAAQRLEPGRNM^73^** | 4( Sp-2/Sp-3/EU-21) | 4 (Sp-2/EU-24/Sp-5/EU-23 |  |
|  | **^70^GRNMWCKIGHTT^81^** | 2 (EU-21/EU-9) | 2 (EU-9/EU-24) | MWCKIGHDRCEE ^1,2,3^ |
|  | **^86^DHDELSMVIPPG^97^** | 1 (EU-9) |  | GYDNLKLEGYYA ^1,7^ |
|  | **^134^VFVDKLHQFICA^145^** | 1 (EU-9) |  | QFICAEHDGHNS ^1^ |
|  | **^150^NRSTISAAHNIS^161^** | 1 (Sp-3) |  |  |
|  | **^166^IYYHHQIDGGNW^177^** | 2 (Sp-3/EU-9) |  | GYYAWLAFLSFS ^7^ |
|  | **^178^FHLEWLRPFFSS^189^** | 1 (Sp-28) | 2 (Sp-5/EU-23) | EWLRPLFSSWLV ^1^ |
|  | **^182^WLRPFFSSWLVL^193^** | 1 (Sp-5) |  |  |
|  | **^226^WSFRTLAAEAQE^237^** | 1 (EU-9) |  |  |
|  | **^230^TLAAEAQERRRA^241^** |  | 1 (EU-11) |  |
|  | **^238^RRRALLAGNHPN^249^** | 1 (EU-9) | 1 (EU-11) | KFPSESRPNVVK ^1,3^ |
|  | **^242^LLAGNHPNVVKP^253^** | 6 (EU-21/EU-9/EU-14/Sp-28) | 5 (EU-9/EU-14/EU-14/EU-11/EU-23) |  |
|  | **^246^NHPNVVKPLALP^257^** | 3 (Sp-3/EU-9/Sp-28) | 1 (EU-23) |  |

a: Broadly neutralizing sera; b: Poor cross-neutralizing sera; c: Isolate use for immunization

1: Vanhee et al. (2011); 2: de Lima et al. (2006); 3 : Oleksiewicz et al. (2000); 4: Meulenberg et al. (1997); 5: Costers et al. (2010); 6: Plagemann, (2004); 7: Zhou et al. (2007)

**Supplementary data 2.2.** Peptides in the ectodomains of the GP4 and GP5 of the EU-24 isolate used in the study and their correspondence with peptides previously described in the literature for the Lelystad strain, prototype of PRRSV-1. Neutralizing peptides are marked in blue and non neutralizing peptides in orange.

| Protein | Amino acid sequence | bSera | pSeraSN | Peptides previously described |
| --- | --- | --- | --- | --- |
| GP4 | **^39^TAAAGFLVLQDI^50^** | 2 (Sp-5/Sp-28) | 5 (EU-24/EU-11/Sp-5/EU-23) | Not described |
|  | **^63^RRKPSQCREAIG^74^** | 1 (EU-24) |  | **ISFGKSSQCREA^1,2^** |
|  | **^67^SQCREAIGTPQY^78^** | 1 (EU-24) |  |  |
|  | **^71^EAIGTPQYVTIT^82^** | 1 (EU-14) | 2 (EU-14/EU-24) |  |
|  | **^75^TPQYVTITANVT^86^** |  | 1 (EU-14) |  |
|  | **^79^VTITANVTDESY^90^** | 1 (EU-14) | 1 (EU-24) |  |
|  | **^83^ANVTDESYLYNA^94^** | 1 (EU-14) | 1 (EU-24) |  |
|  | **^131^FTDYVAHVTQHT^142^** | 3 (EU-21/EU-9/EU-14) |  | Not described |
|  | **^143^QQHHLVVDHIRL^154^** | 3 (Sp-3/EU-21/EU-9) | 1 (Sp-2) | Not described |
|  | **^147^LVVDHIRLLHFM^158^** | 2 (Sp-3/EU-9) | 1 (Sp-2) |  |
|  | **^151^HIRLLHFMTPST^162^** | 2 (Sp-3/EU-9) | 1 (Sp-2) | Not described |
|  | **^155^LHFMTPSTMRWA^166^** | 2 (Sp-3/EU-9) |  | Not described |
|  | **^159^TPSTMRWATTIA^170^** | 1 (EU-9) | 2 (Sp-2/EU-11) | Not described |
| GP5 | **^31^FADGKGDSSTYQ^42^** |  | 1 (EU-24) | ***DSSTYQYIYNLT* ^6^** |
|  | **^35^KGDSSTYQYIYN^46^** | 6  (Sp-2/EU-9/EU-14/  Sp-5/Sp-28) | 3 (EU-14/EU-24) |  |
|  | **^39^STYQYIYNLTIC^50^** | 2 (EU-14) | 3 (EU-14/EU-24) |  |
|  | **^59^SEHFDWAVETFV^70^** | 1 (EU-9) |  | Not described |
|  | **^99^SVTGFYDGRYV^110^** |  | 3 (EU-11/Sp-5/EU-23) | Not described |
| M | **^1^MGGIDGFCFDPT^12^** | 2 (EU-9/Sp-28) |  | **MGGLDDFCNDPI ^1^** |

a: Broadly neutralizing sera; b: Poor cross-neutralizing sera; c: Isolate use for immunization

1: Vanhee et al. (2011); 2: de Lima et al. (2006); 3 : Oleksiewicz et al. (2000); 4: Meulenberg et al. (1997); 5: Costers et al. (2010); 6: Plagemann, (2004); 7: Zhou et al. (2007)
